# A Hospital-based Study of the Prevalence and Usage of Complementary and Alternative Medicine Among Saudi Psychiatric Patients

**DOI:** 10.7759/cureus.5584

**Published:** 2019-09-06

**Authors:** Mohammad Hasan Rajab, Fouad Jabri, Baraa Alghalyini, Leen Raddaoui, Khaled Rajab, Mohammed A Alkhani, Lisa Doraine Holmes, Fahad AlDosary

**Affiliations:** 1 Biostatistics, Epidemiology, and Public Health, College of Medicine, Alfaisal Univerity, Riyadh, SAU; 2 Biostatistics and Epidemiology, College of Medicine, Alfaisal University, Riyadh, SAU; 3 Family and Community Medicine, College of Medicine, Alfaisal University, Riyadh, SAU; 4 Internal Medicine, George Washington University Hospital, Washington, D.C. , USA; 5 Psychiatry, College of Medicine, Alfaisal University, Riyadh, SAU; 6 General Surgery, King Faisal Specialist Hospital and Research Center, Riyadh, SAU; 7 Public Affairs, University of Texas at Dallas, Allen, USA; 8 Mental Health, National Neurosciences Institute, Riyadh, SAU

**Keywords:** complementary and alternative medicine (cam) therapies, psychiatric patients, hospital-based study, cross-sectional study, kingdom of saudi arabia, prevalence

## Abstract

Introduction

Complementary and alternative medicine (CAM) therapies, used singularly or in combination with more conventional therapies, are routinely used by citizens of the Kingdom of Saudi Arabia (KSA) for medical care or seeking wellness. However, the prevalence of CAM therapies among Saudi psychiatric patients is not yet documented. To better understand the importance of CAM in today’s medical field, particularly within the KSA and for psychiatric patients, this descriptive study aims to characterize the use of CAM therapies by a sample of psychiatric patients in the KSA.

Methods

This cross-sectional hospital-based study describes the use of CAM therapies by Saudi psychiatric patients being treated at one of the largest government hospitals in the KSA. Using a pretested questionnaire, adult psychiatric patients waiting for their appointment or during their stay at a government hospital in Riyadh were interviewed regarding the use of CAM therapies after agreeing to participate.

Results

Forty-five adult psychiatric patients agreed to participate in this study. The average age of the participants in the study was 35. Of the participants, 62% were females, 91.1% were outpatients, and 44.4% were diagnosed with depression. Moreover, 82.2% of the participants reported using one or more types of CAM therapies within the past year to address mental illness. Of those who used CAM therapies, 62.2% did so to improve their quality of life; 59.5% did so for treatment, supportive treatment, or both, and 54.1% used CAM therapies to help control symptoms. The most frequent CAM therapies used by psychiatric patients were spiritual therapies such as Quran recitation; body therapies, mainly exercise; and mind therapies, mainly relaxation techniques. These CAM therapies were used mainly as treatments or supportive treatments for depression. Most of the participants who used CAM therapies were satisfied with the use of these therapies (75.7%). Finally, 45.9% of the participants had not discussed the use of CAM therapies with their doctors, primarily because of their reluctance to share private information, especially spiritual matters, with the treating physicians.

Conclusions

The study results suggest a high prevalence of CAM therapy use among Saudi adult psychiatric patients, as well as a high level of satisfaction with such therapies. However, since about half of the patients had not discussed the use of CAM therapies with their physicians, and since some of the CAM therapies may cause unfavorable interactions when used alongside certain medications or medical interventions, healthcare providers should be diligent about inquiring of their psychiatric patients any use of CAM therapies, not only during the initial visit but also during the follow-up visits as well.

## Introduction

Complementary and alternative medicine (CAM) therapies, pursued either singularly or in combination with more conventional therapies, are routinely used by Saudis for medical care or seeking wellness [1]. A study conducted in Western Saudi Arabia revealed that 42 % of families used CAM therapies to treat their children even before seeking medical help [2]. Other studies have determined that within the KSA, spiritual therapies such as prayers and Quran recitation are the most prevalent CAM therapies [3].

Studies have found that patients with psychiatric disorders such as depression or anxiety engage in the use of CAM therapies more often than the general population [4-5]. Overall, the literature search reveals that the prevalence of, and satisfaction with, the use of CAM therapies among adult psychiatric patients in the KSA are not yet documented. A better understanding of the use of CAM therapies among psychiatric patients is likely to improve patient care.

In a study conducted in the United States, only 34% of psychiatric patients reported that they had discussed their use of CAM therapies with their psychotherapist [6]. A similar trend was also reported in Saudi cancer patients [3]. However, it has not been known whether psychiatric patients in Saudi Arabia have discussed the use of CAM therapies with their treating physicians. Such knowledge is important because of the need for all patients to know the potential for unfavorable interactions to occur when CAM therapies are used alongside more conventional therapies [7]. For example, many herbal therapies are safe to use but may still cause unfavorable interactions when used alongside certain medications or medical intervention [8]. 

While the majority of healthcare providers in the KSA have inadequate formal training for working with patients who use CAM therapies, training in alternative medicine in conjunction with conventional medicine has become increasingly available to healthcare providers over the past decade [9].

The purpose of this study was to characterize the use of CAM therapies among adult psychiatric patients during treatment at a major government hospital in the KSA. More specifically, it was to examine the types of CAM therapies being used along with the reasons for their use. Last, but not least, the study also investigated whether psychiatric patients were satisfied with their choice of therapies and whether they had discussed their use of CAM therapies with their healthcare providers.

## Materials and methods

A cross-sectional hospital-based study was conducted among 45 Saudi adult psychiatric patients receiving treatment at one of the largest government hospitals in the KSA, with a total capacity of (1200) beds. The questionnaire used in this study was developed by the principal investigator of this study and his colleagues in their previously published studies [6, 10]. Following institutional review board (IRB) approval, adult psychiatric outpatients awaiting their appointments, as well as inpatients residing at the hospital, were approached to solicit their participation in the study.

After enrollment, the participants were then asked to complete the pretested questionnaire that provided their demographics, their medical diagnoses, type of (and satisfaction with) CAM therapies used, and information on whether participants had discussed their use of CAM therapies with healthcare providers.

Exclusion criteria included patient inability to give informed consent for any reason including dementia; inability to read and/or write; the existence of certain physical limitations, such as neurological disorders or cognitive impairments that prevented completion of questionnaires; and patients less than 18 years of age. Demographic characteristics including age, gender, and marital status were summarized using descriptive statistics as appropriate. Version 25 of the Statistical Package for the Social Sciences (SPSS) (IBM Corp Armonk, NY) was used for data management and statistical analysis.

## Results

Forty-five adult psychiatric patients agreed to participate in this study. The enrollment process took an average of 30 minutes to complete. The mean age for study participants was 35.4 ±1, and ages ranged from 20-64 years. Out of these participants, 91.1% were outpatients, 62% were females, 51.1% were married, and 53.3% had been diagnosed with at least one psychiatric disorder in the past five years. The most often reported diagnoses included depression (44.4%), anxiety (8.9%), obsessive-compulsive disorder (8.9%), and schizophrenia (8.9%). Baseline characteristics of study participants are summarized in Table 1. 

**Table 1:** Baseline characteristics of the study participants (n = 45)

Demographic characteristics	n (%)	Clinical characteristics	n (%)^*^
Gender		Diagnosis	
Male	17 (38)	Depression	20 (44.4)
Female	28 (62)	OCD	4 (8.9)
Age	35.4 ±11.1 (20, 64)^ǂ^	Anxiety	4 (8.9)
Marital status		Schizophrenia	4 (8.9)
Single	15 (33.3)	ADHD	1 (2.2)
Married	23 (51.1)	Eating disorder	1 (2.2)
Divorced	5 (11.1)	Sleep disorder	1 (2.2)
Widowed	2 (4.4)	Insomnia	1 (2.2)
Date of diagnosis (n, %) ^~^		Hallucination	1 (2.2)
≤ 5 years	24 (53.3)	Somatization	1 (2.2)
6-9 years	5 (11.1)	Stress	1 (2.2)
≥10 years	7 (15.6)	Anger issues	1 (2.2)
		Impulse control disorder	1 (2.2)
		Autism	1 (2.2)
		Treatment modality	
		Outpatient	41 (91.1)
		Inpatient	4 (8.9)
^~ ^Missing = 9, ^ǂ ^Mean ± SD (minimum, maximum), ^* ^Missing = 3			

Of those who participated, 82.2% reported using one or more types of CAM therapies within the past year to deal with mental illness. The majority of participants (62.2%) used CAM therapies to improve their quality of life; 59.5% used cam therapies as treatments or for supportive treatment; and 54.1% did so to help control symptoms. The use of CAM therapies by study participants and reason for use in the past 12 months are summarized in Table 2.

**Table 2:** Study participants’ use of CAM therapy in the past 12 months

	n (%)
Used CAM therapies in the past 12 months (N=45)	37 (82.2)
Reasons for using CAM therapies (N=37)	
To improve quality of life	23 (62.2)
Treatment or supportive treatment	22 (59.5)
Symptom control	20 (54.1)
Other purposes	2 (5.40)

Three types of CAM therapies are addressed in the study: spiritual, body, and mind. For all participants who used CAM therapies, at least one type of spiritual therapy was undertaken, such as Quran recitation. Concerning the body therapies, 86.5 % reported using at least one type, mainly exercise (67.6%) and special diet (67.6%). Finally, 75.7% of the participants were using mind therapies, including relaxation techniques (75.7%) and meditation (27%). Types of CAM therapy used by study participants within the past 12 months according to diagnoses are summarized in Table 3.

**Table 3:** Type of CAM therapy used in the past 12 months by diagnosis (n = 37)

Therapy	Diagnosis					
	Depression	Anxiety	Obsessive-compulsive disorder	Schizophrenia	Other	n (%)*
Spiritual						
Quran recitation	17	4	3	3	9	36 (97.3)
Prayer	17	4	3	3	8	35 (94.6)
Other	12	3	2	3	9	29 (78.4)
Body						
Diet, nutrition	13	3	1	1	7	25 (67.6)
Exercise	11	4	3	2	5	25 (67.6)
Topical apps	6	0	0	0	3	9 (24.3)
Traditional Chinese medicine	3	1	1	0	2	7 (18.9)
Other	1	0	0	0	0	1 (2.7)
Mind						
Relaxation	14	4	2	2	6	28 (75.7)
Meditation	6	1	1	0	2	10 (27.0)
Other	3	0	0	0	0	3 (8.1)
^*^Data for one patient was missing						

Most participants (75.7%) reported satisfaction with the therapies they used. Of all 45 participants, 45.9 % had not discussed the CAM therapies they used with their healthcare provider. The reasons provided for not doing so include: (1) unnecessary to do so, (2) dissatisfaction with the therapies, and/or (3) unwillingness to share private information, especially spiritual therapies, with the treating physicians. The study's main outcomes are summarized in Table 4.

**Table 4:** Main outcomes for CAM therapy users (n = 37)

	n (%)^*^
Satisfied with CAM therapy	28 (75.7)
Had not discussed CAM Rx with healthcare provider	17 (45.9)
^*^Data for one patient was missing	

## Discussion

In the past year, spiritual therapies, including prayers and Quran recitation, were the most frequent CAM therapies used by study participants. The second most frequent type was body therapies, including exercise, and special diets. The least frequent type of CAM therapies was mind therapies, mainly relaxation, and meditation. Similar results were recently reported in a cancer patient population in the KSA [3]. Study participants used different types of CAM therapies primarily to improve the quality of life, provide treatment or supportive treatment, or control symptoms.

One of the more notable findings of the study was the fact that nearly half of the study participants reported not discussing the use of CAM therapies with their healthcare providers. This finding was of considerable concern because of the unfavorable interactions that can inadvertently occur when certain types of CAM therapies, such as herbs or dietary supplements, are used alongside some of the more conventional psychiatric medications. For example, using antidepressants alongside Panax ginseng may induce mania in depressed patients, and combining antidepressants with yohimbine (*Pausinystalia johimbe*) may increase the risk of hypertension [7]. Part of the problem, besides the reasons provided by patients for not providing information to physicians about their use of CAM therapies, is that physicians do not always discuss CAM therapy options with their patients before or during the course of treatment. This is important because a significant number of people are using different types of CAM therapy [5].

The standard of care for all patients during their first appointment includes a review of their comprehensive medical history. However, if the patient begins to use or change CAM therapies thereafter, this new development may be missed by the treating doctor. It is for this reason that healthcare providers should inquire at each visit about the patient’s use of CAM therapies [11]. 

Another significant realization garnered from this study interviews was that many Saudi patients tend to view spirituality as an integral part of their daily living activities rather than as only a part of CAM therapies. So it may be that the spirituality aspect of CAM therapies is inadvertently being omitted from patients’ medical histories, including the histories of psychiatric patients. Psychiatrists, in particular, are reputably known to concentrate only on the biological and psychological causes of mental illness during their analysis of patients’ conditions [12]. Yet another recurring issue faced by patients and physicians in obtaining and recording patient information during the course of treatment is that many non-Arabic speaking physicians who gleaned information during the medical visit also lost valuable information during the translation, which can also affect the accuracy of patient records and the effectiveness of patient care [unpublished data]. 

Some limitations that might impact the study findings should be addressed. First, the study was conducted using a cross-sectional, single-site methodology, which can be problematic because of the potential introduction of non-response bias which may have impacted the generalizability of results. Nevertheless, the use of cross-sectional studies continues to be widespread among researchers and has proven to be quite informative and beneficial despite limitations [13]. Second, the sample size was small due to the challenges encountered during the recruitment of psychiatric patients at the hospital who were awaiting appointments or staying there. Lastly, the failure to include certain questions in the survey limited the ability to conduct an in-depth analysis. For example, the study was unable to obtain information on patient knowledge of the potential for unfavorable interactions of some CAM therapies with conventional treatments. Therefore, the results of the present study may have limited generalizability.

Notwithstanding these limitations, this study helps to fill an existing gap in Saudi psychiatric literature. However, the strategies that can be used to improve the generalizability of the findings include surveying several major hospitals with larger sample sizes. 

## Conclusions

The study results suggest that the use of CAM therapies is highly prevalent among Saudi adult psychiatric patients. While two of the main types of therapies (body and mind) are being utilized to nearly the same extent by the study participants; all study participants were engaged in spiritual therapies of some kind. Of all the therapies available, these participants sought Quran recitation, special diet, exercise, and relaxation techniques the most. It would be helpful to explore why these therapies are preferred by patients with the highest percentages with diagnoses in depression, obsessive-compulsive disorder, anxiety, and schizophrenia. The study results also show that many patients do not inform their healthcare providers about their use of CAM therapies. Therefore, as part of a well-rounded and effective treatment plan, healthcare providers should faithfully inquire of patients their use of CAM therapies, not only during the initial visit but also during the follow-up visits as well.
